# Genome Sequence Analysis of Two *Pseudomonas putida* Strains to Identify a 17-Hydroxylase Putatively Involved in Sparteine Degradation

**DOI:** 10.1007/s00284-018-1573-2

**Published:** 2018-09-28

**Authors:** Andrew P. Detheridge, Gareth W. Griffith, David J. Hopper

**Affiliations:** Institute of Biological, Environmental and Rural Sciences, Aberystwyth University, Aberystwyth, Ceredigion, Wales UK

## Abstract

Two strains of *Pseudomonas putida*, Psp-LUP and Psp-SPAR, capable of growth on the quinolizidine alkaloids, lupanine and sparteine respectively, were studied here. We report the isolation of Psp-SPAR and the complete genome sequencing of both bacteria. Both were confirmed to belong to *P. putida*, Psp-LUP close to the type isolate of the species (NBRC14164^T^) and Psp-SPAR close to strains KT2440 and F1. Psp-SPAR did not grow on lupanine but did contain a gene encoding a putative quinolizidine-17-hydroxylase peptide which exhibited high similarity (76%identity) to the lupanine-17-hydroxylase characterised from Psp-LUP.

## Introduction

Removal of the neurotoxic alkaloid lupanine, present in the beans of many (“bitter”) varieties of cultivated lupin (*Lupinus albus*), is required before consumption by humans or livestock. Whilst this can be achieved by prolonged soaking in water, more rapid removal can be mediated via microbial fermentation [1, 2]. A strain of *Pseudomonas* (Psp-LUP [= DH2001]), capable of growth on lupanine as sole source of carbon and nitrogen, was isolated from soil in Poland by Reifer et al. [3], and its use for the removal of lupin alkaloids was suggested. Reifer et al. [3] referred to this organism as “*Pseudomonas lupanini*” but it was not formally described. Later examination of this isolate by Hopper et al. [4] found it to form a green-yellow fluorescent pigment when cultivated on King’s B agar, and to exhibit other cytological and biochemical similarities to *Pseudomonas putida*.

**Fig. 1:**
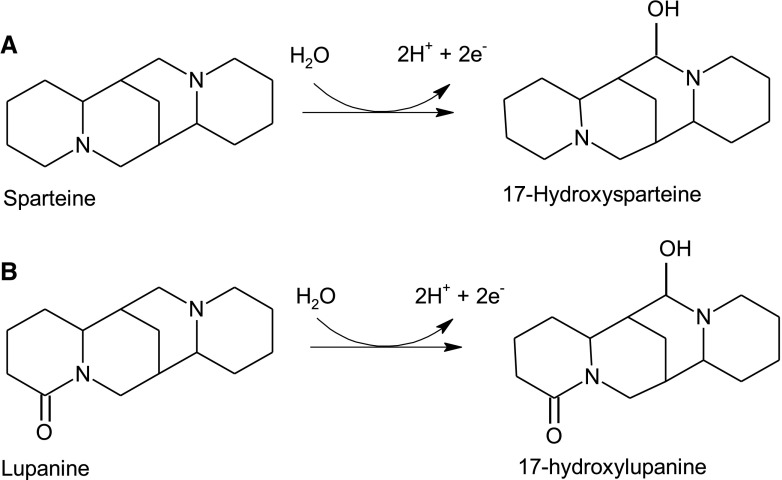
Reactions catalysed by (**A**) sparteine 17-hydroxylase and (**B**) lupanine 17-hydroxylase

Investigation of the mechanism of lupanine degradation by Psp-LUP showed that the initial step in degradation of the fused heterocyclic ring structure containing tertiary nitrogen atoms (Fig. 1) is hydroxylation in the 17- position to give 17-hydroxylupanine [5]. This step is catalysed by an inducible enzyme [6] which has been fully characterized as PQQ (Pyrroloquinoline quinone)-containing haemoprotein [4]. The enzyme is not an oxygenase but acts as a dehydrogenase to give a double bond which is then hydrated to introduce the hydroxyl group. The gene for the enzyme has been sequenced and active enzyme characterised following its heterologous expression [7, 8].

The dominant alkaloid in the South American lupin (*L. mutabilis*) is sparteine, also a quinolizidine alkaloid, which differs from lupanine in lacking an oxygen on carbon 2 (Fig. 1). Unlike lupanine which must be purified from lupin beans, sparteine is commercially available and is used by chemists as a chiral ligand in organic syntheses [9].

It is likely that similar pathways are used for the degradation of these two compounds and to pursue further the bacterial catabolism of these, a second strain *Pseudomonas* (Psp-SPAR) was isolated. As the sequence of the gene for lupanine hydroxylase is known (AJ318095; [7]), it was decided to sequence the genomes of both organisms to see if a similar gene was present in the sparteine-degrading bacterium.

## Materials and Methods

### Isolation of *Pseudomonas putida* (Psp-SPAR)

The organism was isolated from a garden compost heap in Aberystwyth, Wales (52.4156,-4.0591) in 2010 by selective culture on mineral medium containing 50 mM K+/Na + phosphate buffer (pH 7.0) containing 4 ml/L of salts solution [10], with 2 mM sparteine as the sole carbon and nitrogen source at 30 °C. It was capable of growth aerobically in liquid medium with sparteine (0.05%, w/v) as sole carbon and nitrogen sources with a generation time of just over an hour. Under these conditions there was no growth on the related compound lupanine.

Extracts of cells were prepared as previously described [4]. Sparteine oxidation was measured using a Clark-type oxygen electrode in a stirred vessel at 30 °C. Reaction mixtures contained in 3.0 mM of 50 mM phosphate buffer (pH 7.0), 1 mg phenazine methosulphate, 1 μmol sparteine and 0.2 ml of crude cell extract. Products were extracted with dichloromethane and examined by GCMS on an HP-5 MS column (25 m x 0.2 mm x 0.33 µm film) with helium as the carrier gas and ionization by electron impact.

### DNA Preparation and Genome Sequencing

For DNA extraction, cultures were grown on Nutrient Agar (Oxoid Ltd.). A single colony was resuspended in phosphate-buffered saline (pH7.0) and genomic DNA was purified using an equal volume of SPRI beads and resuspended in EB buffer. The beads were washed with extraction buffer containing lysozyme and RNase A, incubated for 25 min at 37 °C. Proteinase K and RNaseA were added and incubated for 5 min at 65 °C.

DNA was quantified using the Quantit dsDNA HS assay in an Eppendorf AF2200 plate reader (triplicate samples). Genomic DNA libraries were prepared using Nextera XT Library Prep Kit (Illumina, San Diego, USA) following the manufacturer’s protocol with the following modifications: 2 ng DNA instead of 1 ng were used as input, and PCR elongation time was increased to 1 min from 30 s. DNA quantification and library preparation were carried out on a Hamilton Microlab STAR automated liquid handling system (Hamilton Robotics, Reno, NV, USA). Pooled libraries were quantified using the Kapa Biosystems Library Quantification Kit for Illumina (Roche, Pleasanton, CA, USA) on a Roche light cycler 96 qPCR machine (Roche, Pleasanton, CA, USA). Libraries were sequenced on the Illumina HiSeq using a 250 bp paired end protocol.

Reads were adapter trimmed using Trimmomatic 0.30 (http://www.usadellab.org/) with a sliding window quality cut-off of Q15 [11]. *De novo* assembly was performed on samples using SPAdes version 3.7 [12] (http://cab.spbu.ru/software/spades/), and contigs were annotated using Prokka 1.11 [13] (http://www.vicbioinformatics.com/software.prokka.shtml).

### Genome Analyses

The genomic average nucleotide identity (ANI) [14] was calculated via *in silico* DNA–DNA hybridization using Kostas Lab web server (http://enve-omics.ce.gatech.edu/g-matrix/) [15]. This estimates the average nucleotide identity using both best hits (one-way ANI) and reciprocal best hits (two-way ANI) between two genomic datasets [16].

## Results

### Genome Assembly

The draft genome of Psp-LUP comprised 6,457,467 bp with average GC content of 62.3% spread over 88 contigs, with an L50 value of 13 (i.e. The longest 13 contigs together constituted half of the total sequence length, with the 13th largest contig having a size of 156,935 bp [= N50]). The draft genome of Psp-SPAR comprised 6,038,563 bp with average GC content of 61.5%spread over 187 contigs, with an L50 of 28 and N50 of 62,376 bp. Details of sequence and strain deposition are given below.

### Identity of Strains

Sequence data from the 16S rRNA locus placed the samples clearly within *Pseudomonas putida* but with only low resolution (data not shown). Therefore, comparison of the genome sequence data for isolates Psp-LUP and Psp-SPAR alongside a range of other genome sequenced *P. putida* strains was undertaken using whole genome analysis. Yonezuka et al. [17] recently used whole genome comparisons to re-evaluate the intraspecific taxonomy of *P. putida* and identified nine sub-groups within the species, in addition to the type isolate. Representatives of these nine groups alongside and the type species confirmed the identity of both organisms as *Pseudomonas putida*.

**Fig. 2:**
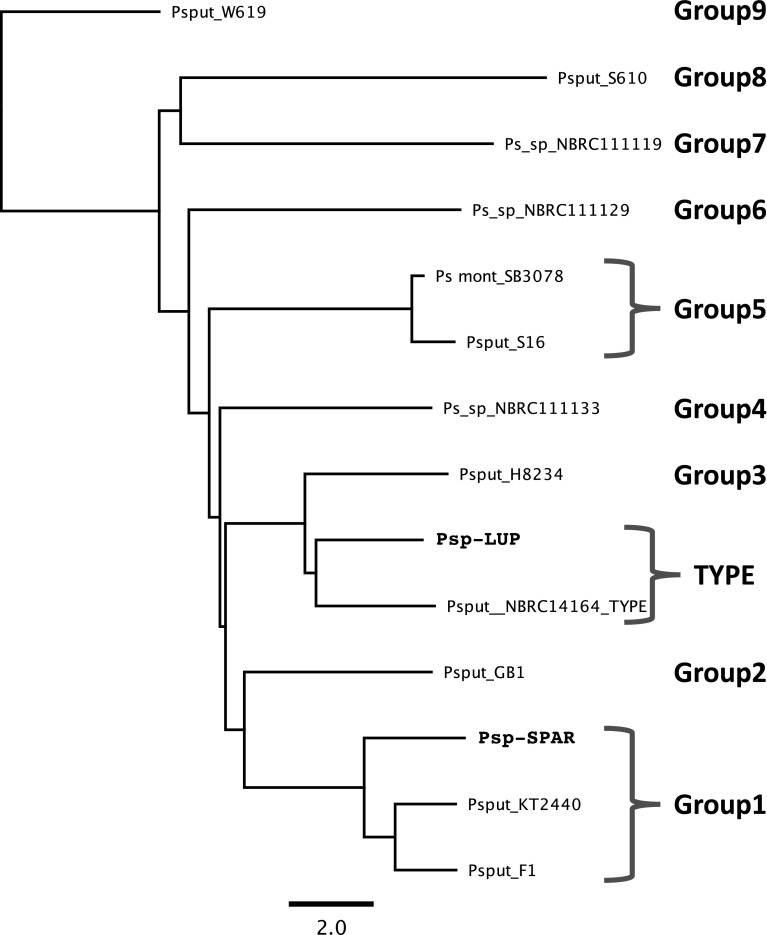
Phylogenetic tree (Neighbour-joining) derived from ANI (Average Nuclotide Identity) analysis of 15 *P. putida* genomes using the grouping designated by Yonezuka et al. (2017). ANI analysis was performed using Kostas Lab web server. Psp-SPAR was clearly placed in group 1, whilst Psp-LUP was closest to NBRC14164^T^, the type specimen for this species (but which was not assigned to any group by Yonezuka et al.). The tree is rooted using the most distant member of the genus (in group 9). Scalebar indicates percentage sequence divergence

Psp-LUP is closely related (ANI similarity value of 95%; Fig. 2) to the type isolate of *P. putida* NBRC14164 (= Stanier strain 90 [biotype A] = A.3.12 = ATCC 12,633 = IAM 1236 [18, 19]) which was isolated from soil [20, 21] whilst Psp-SPAR fell clearly within group1 closest to strain F1 (also isolated from soil; [22]). Psp-LUP was closely related to clinical isolates (found in groups 2,3 and 5; Fig. 2) whereas Psp-SPAR clustered with strains isolated from non-clinical sources [23].

### Presence of the 17-Hydroxylase Gene in Ps Putida (SPAR)

A gene was located in the genome of Psp-LUP with a sequence identical to that for lupanine 17-hydroxylase thus confirming the published data for this enzyme [7, 8]. Examination of the genome of Psp-SPAR revealed a very similar gene coding for a protein of 692 amino acid (c.f. 695 for the lupanine 17-hydroxylase in Psp-LUP). Alignment of the two peptides showed them to be very similar (76% identical residues and 86% conserved/identical). Lupanine 17-hydroxylase has a signal sequence of 26 amino acids for transport of the protein into the periplasm. Analysis of the translated gene from Psp-SPAR using SignalP 4.0 [24] also predicted a signal sequence with a cleavage site in this case between residues 27 and 28. Within the protein sequence was the sequence CSGCH at residues 614–618 (c.f. CGACH, 613–617 for lupanine hydroxylase [GenBank Q934G0.1]), corresponding to the haem-binding consensus sequence of CXXCH. Also conserved within the sequence are eight tryptophan-docking motifs that have been shown to be important in the tertiary structure of a number of PQQ-containing proteins [25, 26], including the quinohaemoprotein dehydrogenase from the bacterium *Comamonas testosteroni* [27], and are also found in lupanine 17-hydroxylase.

### Sparteine Degradation by Psp-SPAR

Extracts of cells of Psp-SPAR, grown on sparteine, contained enzymic activity towards sparteine when incubated with phenazine methosulphate as an electron acceptor. This compound, when reduced, is auto-oxidizable and this results in oxygen uptake as measured in an oxygen monitor. Products were identified by GCMS as 17-hydroxysparteine and 17-oxosparteine from their retention times and mass spectra compared with authentic compounds and the NIST library of spectra. 17-hydoxysparteine was prepared by the cold oxidation of sparteine by acid permanganate. 17-Oxosparteine was prepared by the oxidation of sparteine using alkaline ferricyanide as described by Golebiewski and Spenser [28]. No activity was detected in extracts from succinate-grown cells. Psp-LUP was unable to grow on the medium containing sparteine as sole carbon source.

### Taxonomic Distribution of Lupanine 17 Hydroxylase Genes

**Fig. 3:**
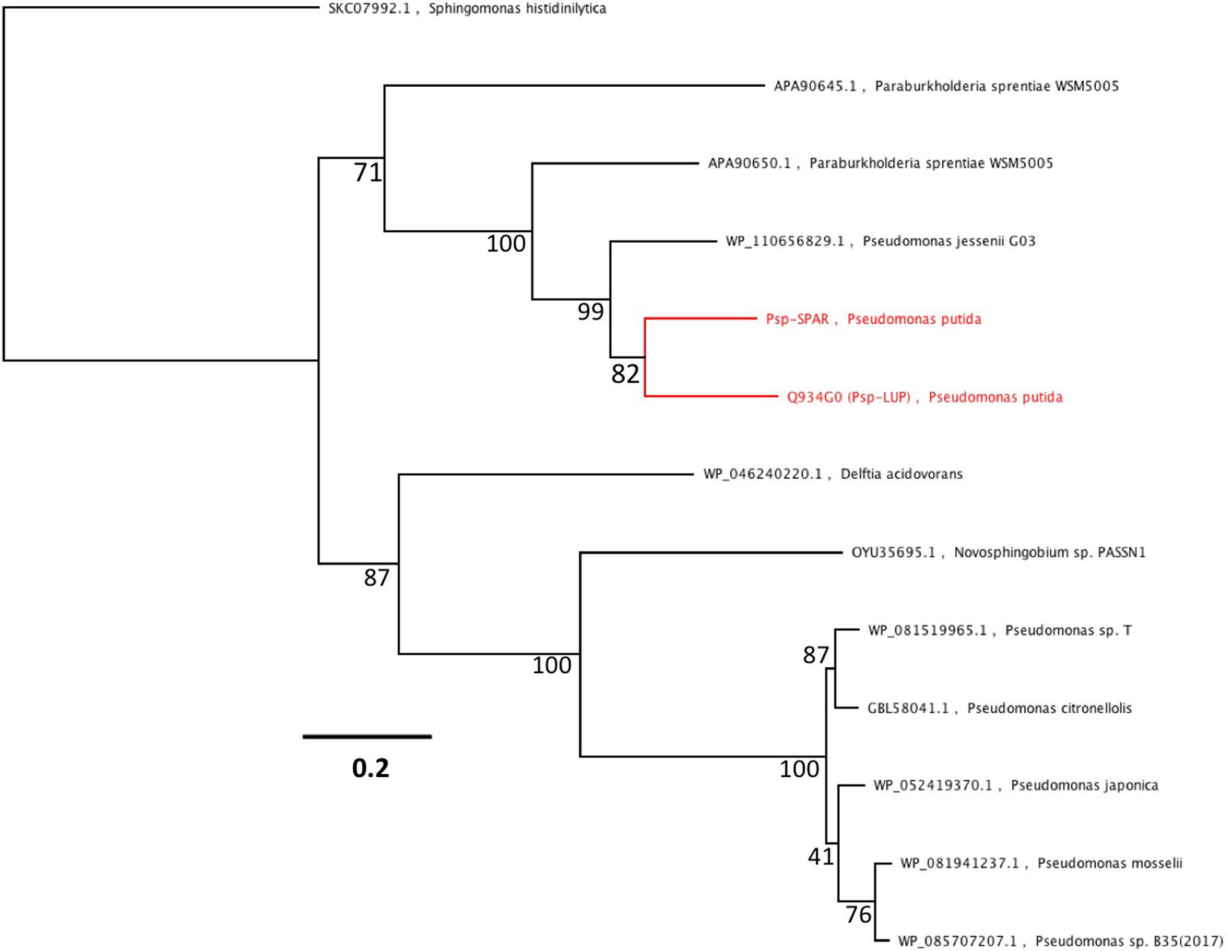
Phylogenetic tree (Maximum Likelihood tree; Le Gascuel substitution model) of the Psp-LUP and Psp-SPAR peptides alongside the most similar proteins identified by BLASTp searches. Numbers at nodes indicated bootstrap values (as % of 1000 replicates).The tree is rooted using the *Sphingomonas histidinilytica* quinohemoprotein ethanol dehydrogenase. Scalebar indicates number of substitutions per site

BLASTp searches with putative peptide sequences from Psp-LUP and Psp-SPAR followed by alignment and phylogenetic reconstruction with the most closely related sequences found on GenBank showed these two sequences to form a distinct cluster along with a putative LUH gene (70% identity to the Psp-LUP peptide) from *Pseudomonas jesennii*, strain GO3 [29], with the next most closely related sequences (from *Paraburkholderia sprentiae* strain WSM5005, plasmid pl2WSM5005 [438 kb]; [30, 31]) being only 62% identical (Fig. 3). These three putative peptides all contained the haem-binding consensus (CXXCH) and the tryptophan docking motifs. BLASTn searches also did not reveal any more closely related sequences. It is noteworthy that the *Paraburkholderia sprentiae* containing the putative LUH sequence was isolated from nodules on the roots of the legume *Lebeckia ambigua* [32]. Like *Lupinus* spp. *Lebeckia* spp. contain high levels of both lupanine and sparteine [33].

There are over 50 sequenced genomes of *P. putida* [17] but the LUH gene is not found in any other strains of this species. This and the fact that the LUH genes within Psp-LUH, Psp-SPAR and *P. jesennii* GO3 are significantly less GC-rich (56.9%, 50.4% and 51.4% respectively) than the genomes in which they occur (all 60-62.3%) suggests that they were acquired by horizontal gene transfer.

## Discussion

Thus, as with lupanine metabolism, the first step in sparteine degradation appears to be its hydroxylation on carbon 17 by a dehydrogenase type of enzyme. The 17-oxosparteine product could be produced by a second dehydrogenase reaction catalysed by the same enzyme. This would be analogous to the oxidation of the methyl group of p-cresol first to p-hydroxybenzylalcohol and then to p-hydroxybenzaldehyde by another dehydrogenase-type hydroxylase, the flavocytochrome p-cresol methylhydroxylase [34]. This first step is consistent with the identification of the possible gene for a sparteine 17-hydroxylase from the genome sequencing. These results showing attack on the 17-carbon are also consistent with the finding by Parmaki et al. [1] of 17-oxolupanine as one of the products of bioconversion of lupanine by *Pseudomonas putida* LPK411.

### Accession Numbers

The complete genome sequences were deposited in the European Nucelotide Archive (https://www.ebi.ac.uk/ena) with the accession number for *Pseudomonas putida* (LUP) of ERX2741807 (PRJEB28091) and for *Pseudomonas putida* (SPAR) of ERX2741806 (PRJEB28090). Genome sequencing was provided by MicrobesNG (http://www.microbesng.uk), which is supported by the BBSRC (grant number BB/L024209/1).Both cultures are deposited at the National Collection of Industrial, Food and Marine Bacteria (NCIMB Ltd., Aberdeen) with accession numbers 15126 (Psp-LUP) and 15127 (Psp-SPAR) (strains deposited but awaiting accession numbers).
